# Editorial

**Published:** 1882-08

**Authors:** 


### EDITORIAL.

An Apology.—These few pages are offered as an apology for our August number, with the following explanation. Both of our associates retired while we were taking our essential summer vacation. Our Dr. Geo. H. Rohé has embarked in his own little boat, the *Medical Chronicle*, of Baltimore, which is a new sprightly monthly for one dollar a year. We say Ro(w)he well and to grand success. Our Dr. Geo. W. Field has returned to London, where we wish him much prosperity. Adieu, dear associates, your labors have been appreciated, and your retiring from the editorial corps of the Independent Practitioner is regretted.

The accumulated business during our absence will not permit us to do justice to the journal at present, but we promise our readers and advertisers ere the close of this year to make amends.

